# High WAVE3 expression correlates with proliferation, migration and invasion in human ovarian cancer

**DOI:** 10.18632/oncotarget.17141

**Published:** 2017-04-17

**Authors:** Jin Lu, Su-Li Wang, Ying-Chun Wang, Yi-Nan Wu, Xi Yu, Wan-Zhou Zhao, Jin-Hua Wang

**Affiliations:** ^1^ Department of Gynecological Oncology Surgery, Jiangsu Cancer Hospital, Jiangsu Institute of Cancer Research, Nanjing Medical University Affiliated Cancer Hospital, Nanjing 210036, China; ^2^ The Nanjing Han & Zaenker Cancer Institute, OG Pharmaceuticals, Nanjing 210036, China; ^3^ Jinling Hospital, Nanjing University, Nanjing 210036, China

**Keywords:** ovarian cancer, WAVE3, cell motility, MMPs

## Abstract

**Background:**

Wiskott-Aldrich syndrome verprolin-homologous (WAVE) 3, a member of the WASP/WAVE family of proteins, plays a critical role in cell motility and acts as an oncogene in some human cancers, but no sufficient information available to illustrate its involvement in ovarian cancer tumorigenesis and progression.

**Methods:**

The expression of WAVE3 in human ovarian cancer and normal tissue was analyzed by immunohistochemistry. WAVE3 gene and protein expression in different human ovarian cancer cell lines was tested by RT-PCR and western blotting. Stable cells of WAVE3-knockdown in SKOV3 cells or transfected high expression in A2780 cells were constructed. The WAVE3 expression and its correlation with MMPs, p38 MAPK and other factors were studied. The relationship between WAVE3 and oncogenicity *in vivo* was also evaluated by nude mice xenograft model.

**Results:**

Immunohistochemistry staining showed the highest WAVE3 expression in ovarian cancer metastases, high in ovarian cancer and weak in normal. In different cell lines, SKOV3 cells showed the highest WAVE3 expression, A2780 cells expressed the lowest. Elevated WAVE3 expression in A2780 cells promoted proliferation and decreased apoptosis, increased the cell number in G2/M phase and promoted migration significantly. Correspondingly, knockdown of WAVE3 in SKOV3 cells showed opposite effects. The WAVE3 expression showed positive correlation with MMPs, NF-κB, COX-2, VEGF and phospho-p38 MAPK, but not p38. The high expression of WAVE3 promoted tumorigenesis *in vivo*.

**Conclusions:**

Our results suggested that WAVE3 may be pivotal in ovarian cancer cell motility, invasion and oncogenesis, which might be related with MMPs production and p38 MAPK pathway.

## INTRODUCTION

Ovaries are located in the depth of the pelvic. Ovarian cancer is one of the most common gynecological malignancies with high mortality rate of the patients due to its easy invasion and metastasis [1]. Lack of early stage signs and symptoms results in that approximately 70% of patients are diagnosed at advanced stage [2], which raised challenge to the subsequent therapy. Clinical staging and histopathological criteria have mainly been used to predict prognosis of patients. Though the treatment methods have been improved significantly, patients with advanced ovarian cancer still lack effective therapy.

Ovarian cancer invasion and metastasis are known as a complex and multi-step cell progresses and associated with many interacting proteins expression, and cellular migration plays a critical role for the ability of tumor cells to metastasize locally and to distant sites [3]. Wiskott-Aldrich syndrome verprolin-homologous (WAVE) 3, together with WAVE1 and WAVE2, belongs to the Wiskott-Aldrich syndrome family proteins (WASPs), which are structurally related proteins and play central roles in multiple cellular processes, including cell shape, motility, cytokinesis as well as cancer cell invasion [4–6]. WAVE3, in particular, is critical for the motility and invasion of cancer cells [7, 8] and has been demonstrated to be upregulated in breast cancer [9], prostate cancer [10], hepatocellular carcinoma [11] and other human cancers [12]. Its overexpression in tumor tissues is associated with the aggressive cancer progression and cancer metastasis [13, 14]. But colorectal cancer patients with high expression of WAVE3 had a better prognosis, including early TNM stage, non-lymph node metastasis and non-distant metastasis [12]. Though WAVE3 particularly, have been proved that might act as an oncogene in some human cancers [12, 14–17], but to the best of our knowledge, there is no sufficient information available to illustrate its involvement in ovarian cancer tumorigenesis and progression.

The progression of ovarian cancer is associated with various genes and proteins. Matrix metalloproteinases (MMPs) have been proved to be involved in cell growth, angiogenesis [18, 19], as well as the degradation of extracellular matrix (ECM), which is a crucial step in tumor invasion and metastasis. Numerous studies have shown that the inhibition of MMPs activity or expression can be a potential way for the prevention of metastasis [20, 21]. Some previous studies have indicated that WAVEs could upregulate MMPs [22] [23] [8, 24]. But it is puzzling that in different type or stage of tumor, some WAVEs act as either enhancers or suppressors of cancer malignancy [24–26]. There is strong evidence showing that WAVE3 is capable of activating the p38 MAPK pathway by Rac [27–29]. Depending on the type of cancer, the function of p38 MAPK might be quite different. In prostatic neoplasia or melanoma, p38 MAPK can positively regulate proliferation [30, 31]. Interestingly, the activated p38 MAPK would emerge as tumor suppressor in various cancers by negative regulation of cell cycle progression, induction of apoptosis and influence of other factors [32–34]. However, the expression pattern, the oncogenic effect of WAVE3 and its correction with MMPs, p38 MAPK and other potential factors in ovarian cancer are still not entirely clear.

To address this problem, we explored the expression of WAVE3 in human primary ovarian cancer, ovarian cancer metastases, the normal ovary tissues, and different human ovarian cancer cell lines. The WAVE3-highest and lowest expression cells were further selected to study the role of the WAVE3 in proliferation, migration and invasion of ovarian cells using *in vitro* systems. Furthermore, we evaluated the relationship between WAVE3 and oncogenicity of the ovarian cancer cells in nude mouse xenograft model *in vivo*.

## RESULTS

### WAVE3 expression is closely related to metastasis in human ovarian tumors

**Figure 1:**
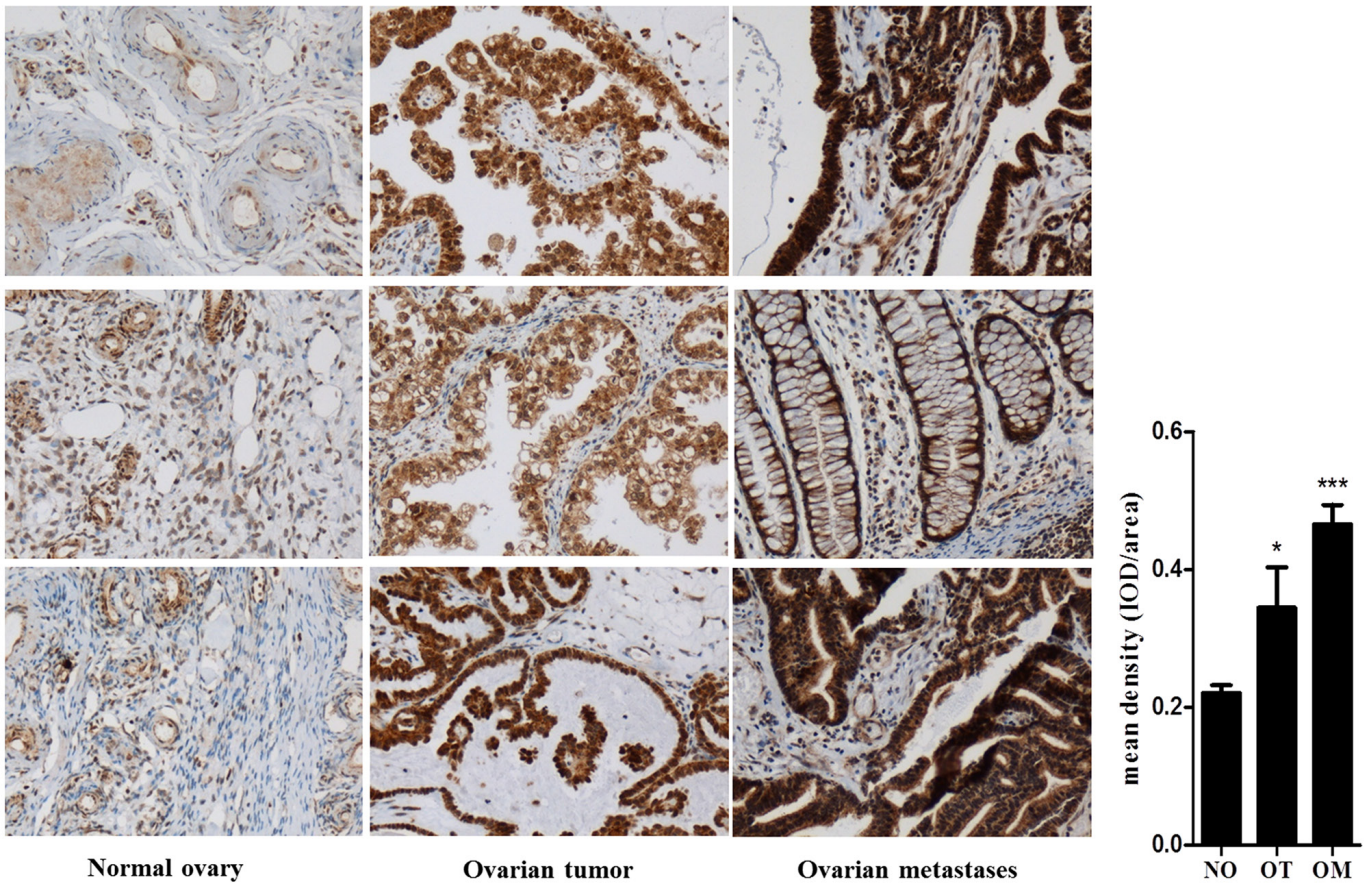
WAVE3 expression was upregulated in human ovarian cancer and ovarian cancer metastases Expression of WAVE3 was examined by IHC in primary ovarian tumor (OT), ovarian cancer metastases (OM) and normal ovarian (NO) tissues from endometrial carcinoma after surgery (n=20). The statistical analysis results of IHC were showed as relative integrated optical density (IOD) which was analyzed by image plus 6.0. (mean ± SD, n = 20, *p < 0.05, ^***^p<0.001 versus NO group). NO: normal ovary group, OT: ovarian tumor group, OM: ovarian metastases group. The specimens were reviewed and graded by two independent pathologists who were blinded to the sample groups.

Patients’ characteristics including age, FIGO stage, histology, FIGO grade and metastasis were demonstrated in Table 1. To verify the relationship between WAVE3 and tumor metastasis, the expression of WAVE3 was examined by IHC in ovarian cancer, advanced ovarian cancer and normal ovarian tissues from endometrial carcinoma after surgery. The results showed weak or negative staining in normal ovary tissues, while different intensity of staining was detected in ovarian cancers (p < 0.05 *vs* normal ovarian tissues). In ovarian tumors with metastasis, the expression of WAVE3 was dramatically higher than that in normal ovary control (p<0.001) (Figure 1).

**Table 1:** Patients’ characteristics (n=60)

Groups	Healthy control	Ovarian cancer	Advanced ovarian
	*(n=20)	(n=20)	cancer (n=20)
Age, median (range)	56.55	53.45	53.30
	(42-72)	(27-70)	(45-78)
FIGO Stage, n (%)		I, 5 (25%)	I, 3 (15%)
		II, 0 (0%)	II, 3 (15%)
		III, 14 (70%)	III, 12 (60%)
		IV, 1 (5%)	IV, 2 (10%)
Histopathology, n (%)		Serous, 18 (90%)	Serous, 16 (80%)
		Clear cell, 1 (5%)	Clear cell, 1 (5%)
		Endometrioid, 1 (5%)	Endometrioid, 2 (10%)
			Mixed, 1 (5%)
FIGO grade, n (%)		1, 0 (0%)	1, 1 (5%)
		2, 4 (20%)	2, 4 (20%)
		3, 16 (80%)	3, 15 (75%)
Metastasis, n (%)			Pelvic floor, 7(35%)
			Intestinal tube, 7(35%)
			Right upper lung, 1(5%)
			Spleen, 1(5%)
			Gastric wall, 2(10%)
			Vaginal stump, 2(10%)

* Normal ovarian tissue from endometrial carcinoma after surgery

### Different expression of WAVE3 in five types of human ovarian cancer cell lines

**Figure 2:**
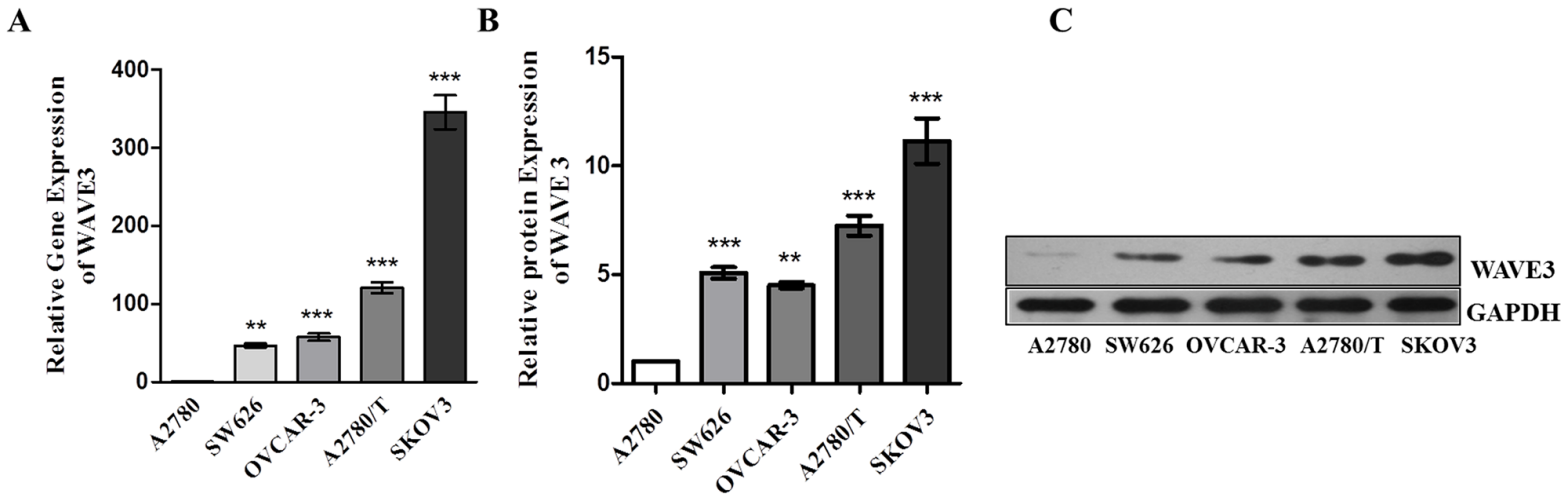
The expression of WAVE3 was compared in five types of ovarian cancer cell lines **(A)** Relative gene expression to GAPDH of WAVE3 in cells A2780, SW626, OVCAR-3, A2780/T and SKOV3 (n=3) which was tested by quantitative real-time PCR. **(B-C)** Relative protein expression to GAPDH of WAVE3 in cells A2780, SW626, OVCAR-3, A2780/T and SKOV3 (n=3) which was tested by Western blotting. (mean ± SD, ^*^p < 0.01, ^***^p<0.001 versus A2780). All immunoblot and real-time PCR analyses performed in the current study were representatives of three independent experiments.

Ovarian cancer cell lines A2780, A2780/T, SW626, OVCAR-3 and SKOV3 were cultured, respectively, and the expression of both WAVE3 mRNA and protein in cells were analyzed. The results showed that although they were all belong to ovarian cancer cells, different cell lines presented various WAVE3 expression levels, of which SKOV3 cell expressed the highest, while A2780 cell expressed the lowest WAVE3 mRNA and protein level (Figure 2). As a result, A2780 cell together with SKOV3 cell were selected reasonably to investigate the role of WAVE3 in the invasion and metastasis of ovarian cancer cells.

### Establishment and the growth characteristic of stable cells

**Figure 3:**
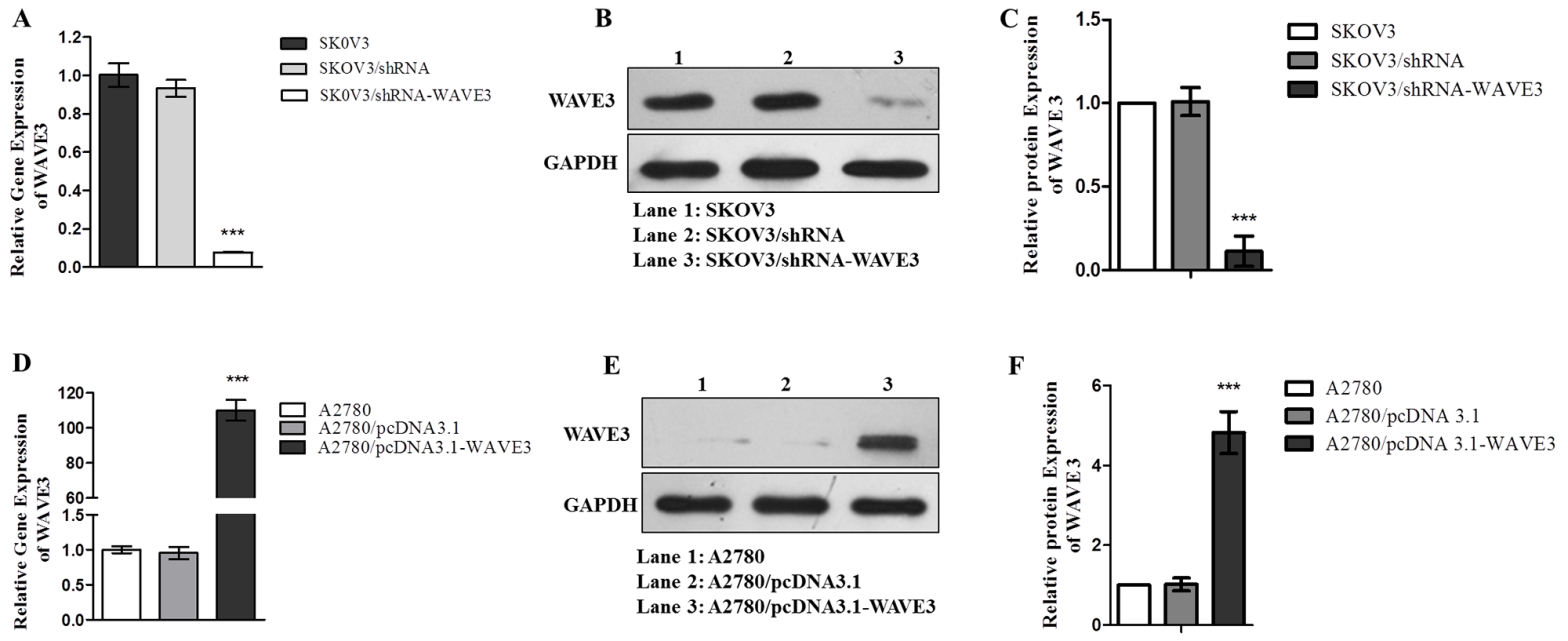
Stable cells SKOV3/shRNA-WAVE3 and A2780/pcDNA 3.1-WAVE3 were constructed SKOV3 cells were transduced with human WAVE3 shRNA lentiviruses or non-targeting control lentiviruses. Six hours later, after selected by 300 μg/mL G418, the expression of WAVE3 mRNA **(A)** and protein **(B-C)** were detected. Stable WAVE3 high-expression A2780 cells were generated by transduced with human pcDNA 3.1 - WAVE3 lentiviruses and selected by G418 (300 μg/mL). The expression of WAVE3 mRNA **(D)** and protein **(E-F)** were analyzed by quantitative real-time PCR and Western blotting, respectively (n=3). (mean ± SD, ^***^p < 0.001 versus SKOV3 or A2780). The housekeeping control GAPDH gene and protein was used to normalize the expression of the WAVE3 gene and protein. All immunoblot and real-time PCR analyses performed in the current study were representatives of three independent experiments.

After transfected with shRNA targeting to WAVE3 and selected with G418, the stable cell SKOV3/shRNA-WAVE3 which low expressed WAVE3 was constructed. The effect of transfection efficiency was proved by the analysis of WAVE3 expression in the cell, comparing with the primary and negative control cells (Figure 3A-3C). In similar way, the stable WAVE3-high expressed cell A2780/pcDNA3.1-WAVE3 was generated (Figure 3D-3F).

### The role of WAVE3 in the cell survival, cell cycle distribution and apoptosis of stable cells

**Figure 4:**
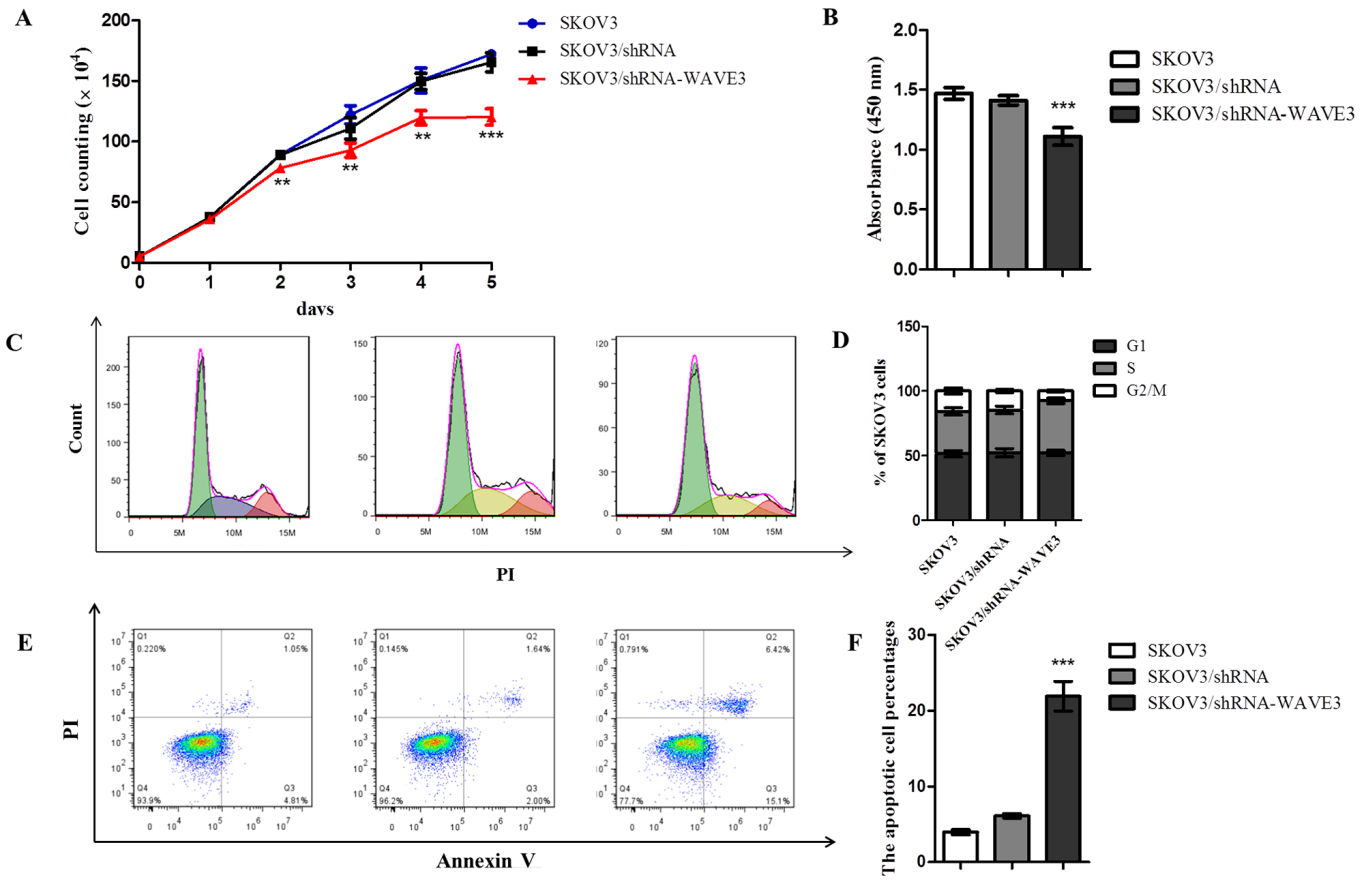
Effect of WAVE3 on the growth, cell cycle distribution and apoptosis of SKOV3 cells **(A)** Growth curve of SKOV3/shRNA-WAVE3 cells with WAVE3 low expression was analyzed in 5 consecutive days. Cell numbers were counted and compared in SKOV3/shRNA-WAVE3 cells and its control cells (n=5). **(B)** In 5^th^ day, cells were incubated with CCK8 solution, and the absorbance at 450 nm was recorded. Cell cycle **(C-D)** and apoptosis **(E-F)** of three types cells were assayed by FCM in day 4 and day 5, respectively (n=5). (mean ± SD, ^*^p < 0.01, ^***^p < 0.001 versus SKOV3). All the experiments were performed in triplicate.

**Figure 5:**
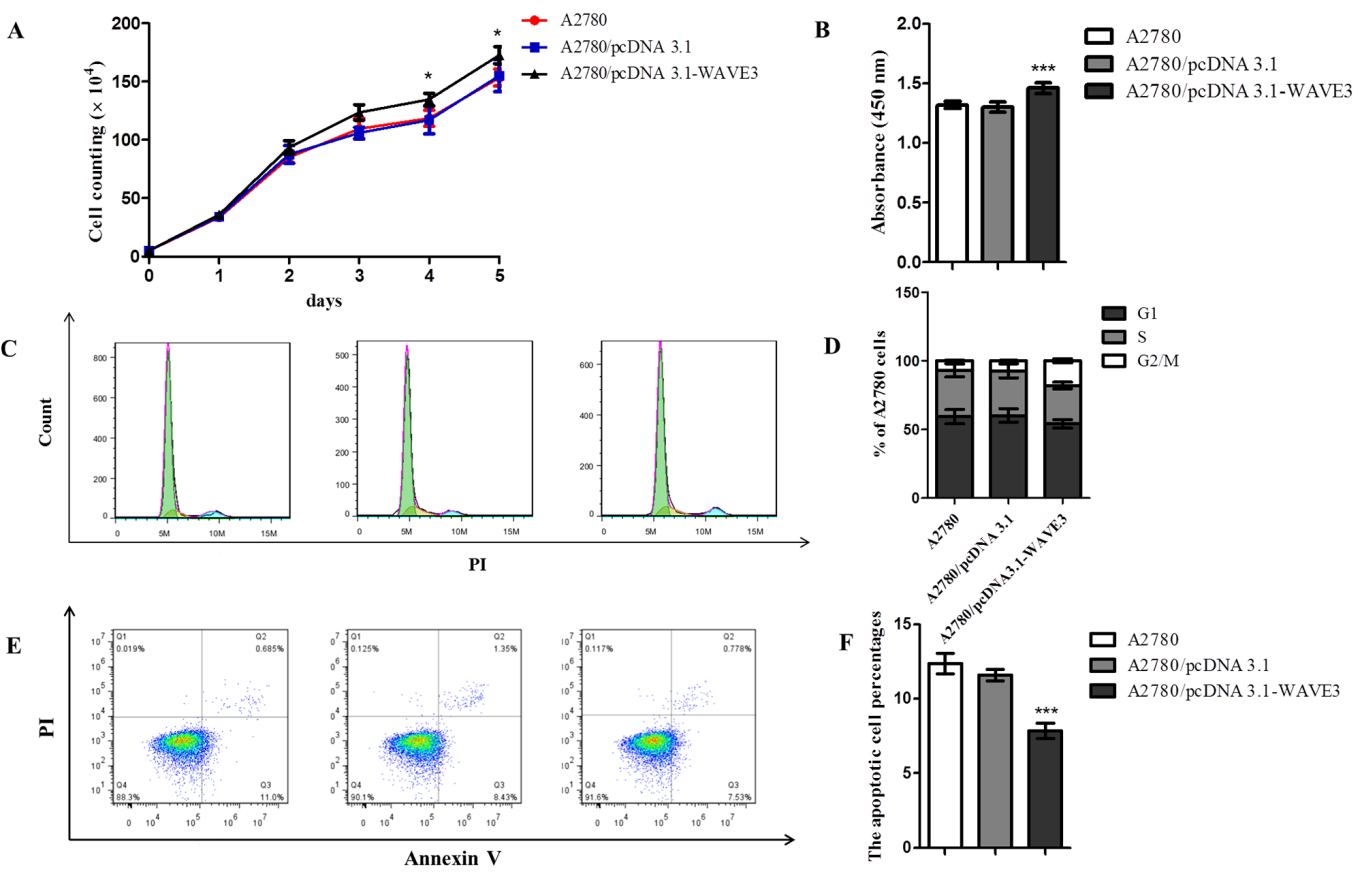
Effect of WAVE3 on the growth, cell cycle distribution and apoptosis of A2780 cells **(A)** Growth curve of A2780/pcDNA3.1-WAVE3 cells with WAVE3 high expression is analyzed in 5 consecutive days. Cell numbers were counted and compared in A2780/pcDNA3.1-WAVE3 cells and its control cells (n=5). **(B)** In 5^th^ day, cells were incubated with CCK8 solution, and the absorbance at 450 nm was recorded. Cell cycle **(C-D)** and apoptosis **(E-F)** of three types cells were assayed by FCM in day 4 and day 5, respectively (n=5). (mean ± SD, ^*^p < 0.05, ^***^p < 0.001 versus A2780). All the experiments were performed in triplicate.

Cell proliferation was observed in 5 consecutive days and the results indicated that WAVE3 showed significant role in cell growth. SKOV3/shRNA-WAVE3 owned a lower growth rate than its control cells in five days (Figure 4A-4B). Correspondingly, A2780/pcDNA3.1-WAVE3 cells showed higher proliferation rate compared to A2780 and empty vector control (p<0.001) (Figure 5A-5B). Besides, cell cycle distribution and apoptosis analysis indicated that WAVE3 gene silence in SKOV3 cells induced cell apoptosis and increased significantly in the percentage of cells in S phase compared to parental cells with a corresponding decrease in G2/M phase. There was no significant difference between two cells in G0/G1 phase (Figure 4C-4F). In A2780 cells part, compared to its parental cells, WAVE3 high-expressed A2780 cells showed lower cell apoptosis rate. And there was an obvious decrease observed in the percentage of the cells in S phase with a corresponding increase in G2/M phase (Figure 5C-5F). There was no significant difference between the two cells in G0/G1 phase.

### WAVE3 enhances the cell migration potential of ovarian cancer cells

**Figure 6:**
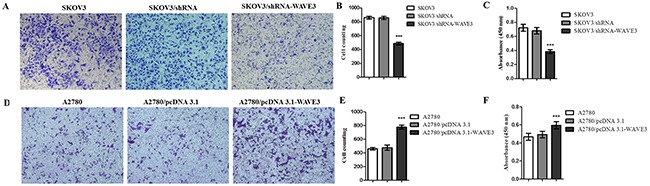
Effect of WAVE3 on cell migration in SKOV3 and A2780 cells The migration of SKOV3 cells infected with human WAVE3 shRNA virus (SKOV3/shRNA-WAVE3) or siControl (SKOV3/shRNA), or A2780 cells transfected by human pcDNA3.1-wave3 (A2780/pcDNA3.1-WAVE3) lentiviruses or siControl (A2780/pcDNA3.1) were examined by transwell migration assay. Equal amount of 1 × 10^5^ cells suspended in serum-free medium were added to the upper chamber, and medium containing 10 % FBS was added to the lower chamber. After incubation in 37°C for 24 h, in which condition the WAVE3 expression had no effect on cell proliferation as presented in Figure 1, cells that migrated to the lower side from the upper chamber were fixed, stained with hematoxylin **(A, D)** and counted **(B, E)**. After migrated cell counting, 200 μL DMSO was added in each wells, and the absorbance at 450 nm was recorded **(C, F)**. (n=3, magnification, 200 ×). mean ± SD, ^***^p < 0.001 versus SKOV3 or A2780. All the experiments were performed in triplicate.

Cancer cells are always associated with malignant properties, such as migration and invasion. In purpose of studying the role of WAVE3 in invasion and metastasis capacity of ovarian cancer cells, WAVE3 was either overexpressed in A2780 cells or depleted in SKOV3 cells, and transwell assay was used for this research purpose. As showing in Figure 6A-6C, the cell migration potential was significantly weakened in SKOV3/shRNA-WAVE3 cells, while dramatically elevated by 2 fold of the control in A2780/pcDNA3.1-WAVE3 cells (Figure 6D-6F). Taken together, these data demonstrated that WAVE3 promotes cell migration in ovarian cancer cells.

### Expression of invasion and metastasis related signaling pathway in ovarian cancer cells

**Figure 7:**
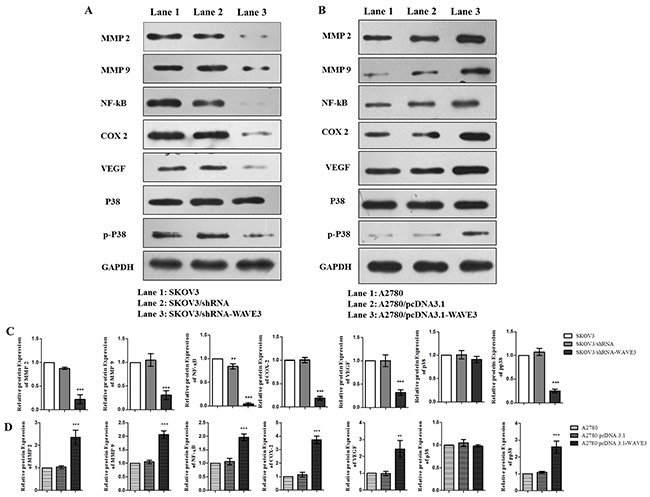
Effect of WAVE3 on invasion and metastasis related signaling pathway in SKOV3 and A2780 cells Total cell lysates were subjected to immunoblot analysis with specific antibodies. Protein expression levels were normalized relatively to that of GAPDH (n=3). mean ± SD, ^*^p < 0.01, ^***^p < 0.001 versus SKOV3 **(A, C)** or A2780 **(B, D)**. All immunoblot analysis performed in the current study are representatives of at least three independent experiments.

Cell samples were subjected to western blotting analysis, and expression of invasion and metastasis related proteins were evaluated in the SKOV3/shRNA-WAVE3 cells, A2780/pcDNA3.1-WAVE3 cells and their parental cells. The results confirmed that the MMP2, MMP9, NF-κB, COX-2, VEGF and P-p38 proteins in the SKOV3/shRNA-WAVE3 cells were all down-regulated (Figure 7A, 7C). Unlikely, the expression of the proteins in A2780/pcDNA3.1-WAVE3 cells showed the opposite trend (Figure 7B, 7D). The expression of p38 in both cells showed no significant changes.

### WAVE3 enhances tumorigenesis of ovarian cancer cells in *vivo*

**Figure 8:**
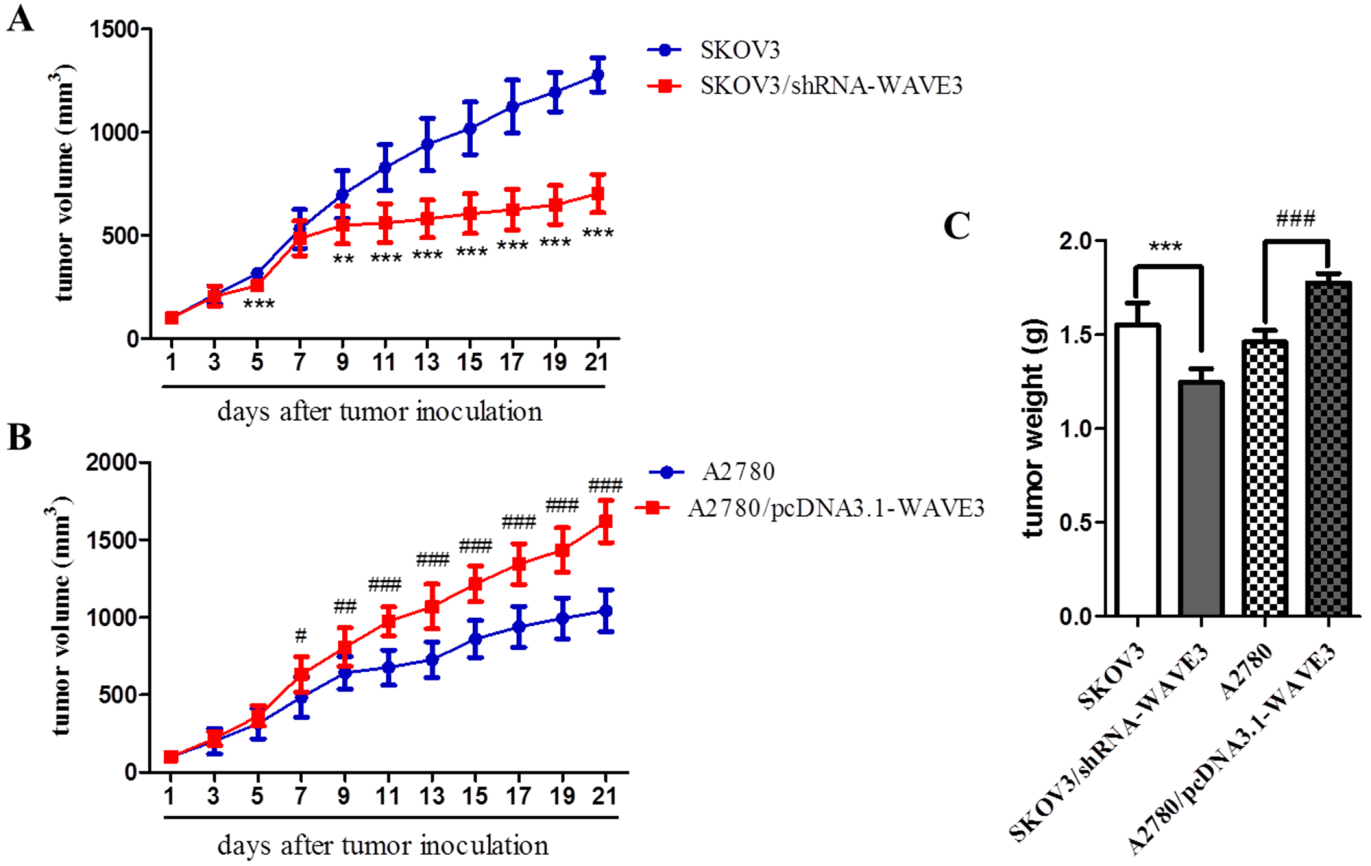
Tumorgenicity of SKOV3/shRNA-WAVE3 and A2780/pcDNA3.1-WAVE3 xenograft model in athymic nude mice **(A-B)** Tumor growth curve after cell inoculation of SKOV3/shRNA-WAVE3 and A2780/pcDNA3.1-WAVE3, respectively. **(C)** Tumors in the right armpit of female nude mice were removed and weighted on the 21th days after implantation. (n=8) (mean ± SD, ^*^p < 0.01, ^***^p < 0.001 versus SKOV3, ^#^p < 0.05, ^##^p < 0.01, ^###^p < 0.001 versus A2780).

To explore the role of WAVE3 in tumorigenicity of ovarian cancer cells in *vivo*, SKOV3/shRNA-WAVE3 cells that expressed low level WAVE3 stably and A2780/pcDNA3.1-WAVE3 cells which is WAVE3 high-expression, together with their primary cells were delivered as cell suspensions and injected subcutaneously into the right armpit of BALB/c athymic nude mice. The dissemination of tumors and tumor volumes were measured and calculated. The results demonstrated that SKOV3 cells and A2780 cells implanted into the mice form tumors rapidly at the site of injection, and tumor volume was increased gradually (Figure 8A, 8B). By contrast, comparing to SKOV3 cells, SKOV3/shRNA-WAVE3 cells xenograft models showed lower growth rate, reflected in smaller tumor volume and weight comparing to SKOV3 cells (Figure 8A, 8C). Conversely, higher growth rate, larger tumor volume and weight were observed in A2780/pcDNA3.1-WAVE3 cells xenograft models, comparing to A2780 cells (Figure 8B, 8C).

## DISCUSSION

According to the National Cancer Institute's (NCI) 2014 statistics, ovarian cancer is the fifth leading cause, but the most prominent one in industrialized countries, of cancer death in women. Cancer invasion, which is started and maintained by signaling pathways that control cytoskeletal dynamics in tumor cells [35], presents a major hurdle in cancer therapy. It is also the first step of cancer metastasis, suggesting that effective inhibition of tumor cells invasion can also effectively inhibit tumor metastasis [36]. Therefore, suppression of tumor metastasis and identification of the relevant molecular markers may facilitate early diagnosis, effective treatment and accurate prognosis assessment.

The association of WAVE3 expression with pathological features of patients with ovarian cancer was explored in our study. WAVE3 expression in ovarian cancer is obviously higher than that in normal ovary. Ovarian metastases showed the highest WAVE3 expression level. Importantly, the immunohistochemical analysis of ovarian cancer samples showed that WAVE3 protein expression level correlated with tumor grade.

In order to acquire more cogent results, the study was started by screening out the WAVE3-lowest and highest expression ovarian cancer cell lines, A2780 and SKOV3, respectively. To explore the effect of WAVE3 in the biological features of the two cell lines more fully, siRNA interference targeting at WAVE3 was used to silent the high expressed WAVE3 in SKOV3. Reversely, plasmid pcDNA3.1-WAVE3 was transfected in A2780 cells to make WAVE3 overexpression. In a follow-up experiment, cell growth curves, cell cycle and apoptosis of four types cells, together with the corresponding negative control cells, were compared carefully. The results indicated that knockdown WAVE3 weakened the proliferation ability of SKOV3 cells significantly, elevated WAVE3 in A2780 cells promoted cell proliferation obviously. Moreover, cell cycle and apoptosis analysis showed that the percentage of G2/M in WAVE3-high expressed A2780 cells was higher than that in A2780 cells, the percentage of S phase was lower than that in A2780 cells in corresponding phases. Conversely, decreased WAVE3 expression in SKOV3 cells reduced the percentage of G2/M and raised the percentage of S phase. According to widely held view, cancer refers to abnormal growth and is characterized by uncontrolled proliferation of cells despite restriction of nutrients and space [37]. Cancer cells have unlimited replication potential *via* the upregulation of telomerase expression [38]. In this part, the result indicate that WAVE3 may play a role in the maintenance of the great potential ability of reproductive activity in ovarian cancer cells, which is the foundation of tumor formation, by the influence of cell cycle and apoptosis of ovarian cancer cells.

Cancer invasion is the first step of cancer metastasis [35], suggesting inhibition of tumor cells invasion could be able to prevent cancer metastasis. Cell motility and invasion abilities are critical components for carcinogenesis, which require highly coordinated regulation of actin dynamics within the cell [39]. The proteins of the WASP/WAVE family have been reported to influence this process and to be implicated in metastasis [40]. Having shown the effect of WAVE3 in cell growth in ovarian cancer cells, we next examined if there is a correlation between WAVE3 and the invasive ability of ovarian cancer cells. In the present part, we observed that knockdown of WAVE3 could inhibit the migration and invasion of SKOV3 cells. Furthermore, the elevated WAVE3 in A2780 cells increased the number of cells that migrated through the basement membrane significantly. All of these suggested that WAVE3 had a promotion effect of invasion in human ovarian cancer cells.

Numerous studies have shown that the inhibition of MMPs activity or expression could be a potential target for the prevention of metastasis [20, 21]. As the member of MMPs family, the role of MMP-2 and MMP-9 in prostate cancer has been extensively studied [41], there are still multiple evidence that shed light on the MMPs and their impact on ovarian cancers [42].

MMPs expression is primarily regulated at the transcriptional level. Most MMPs are normally expressed at low level in tissues, and only induced when the extracellular matrix is remodeled. In the present study, the role of MMPs in ovarian cancer cell invasion was investigated following WAVE-3 knockdown or overexpression. Evaluation of the expression of different MMPs revealed relatively low expression of MMP-2 and MMP-9 after silencing of WAVE-3 in SKOV3 cells. Inversely, elevated WAVE-3 expression in A2780 cells increased the protein level of MMP-2 and MMP-9 relatively. Results of our current study suggested that MMP-2 and MMP-9 may play an important contributory role in cellular invasion and movement, after the remodeling expression of WAVE-3.

As a transcription factor, NF-κB binds to the MMP-9 promoter [43]. It is also involved in cell proliferation, inflammation, and tumor invasion and metastasis, by inducing tumor cell epithelial mesenchymal transformation [44] and upregulation of angiogenic factors expression [45]. Some previous studies showed that downregulation of COX-2 expression could inhibit tumor invasion and metastasis [46]. Besides, p38 MAPK is a downstream effecter of WAVE3 [8], WAVE3-p38 MAPK signaling contributes to the metastatic potential of breast cancer cells [9]. Ovarian cancer is a richly vascularized tumor. VEGF expression is a crucial, early event in ovarian carcinogenesis and associated with tumor growth and aggression, as well as poor survival [47]. Basically through promoting tumor angiogenesis and enhancing the vascular permeability, VEGF owns the key importance in the pathophysiology of the disease. VEGF enhances vascular permeability, a process in which p38 MAPK pathway has been implicated as an essential mediator [48]. VEGF regulates ovarian cancer invasion and migration *in vitro* through VEGFR-mediated secretion and activation of MMP-2, MMP-7, and MMP-9 [49]. Results in this part indicated that WAVE3 inhibition induced the decreased expression of COX-2, VEGF, NF-κB and p38 MAPK in SKOV3 cells, while elevated WAVE3 resulted in the relatively high expression of COX-2, VEGF, NF-κB and p38 MAPK in A2780 cells. Until now, all above results reminder us there is a complex cross talk between WAVE3 and factors related to tumor invasion and metastasis. Our previously study have showed that docosahexaenoic acid (DHA) could downregulate the expressions of WAVE3, vascular endothelial cell growth factor, and MMP-9, which negatively correlated with cell invasion and metastasis *in vitro* and in xenograft model of zebrafish *in vivo* [46].

Together these findings provide evidence that WAVE3 is an important factor in ovarian cancer progression by contributing to tumor cell motility and invasion. Our results demonstrated that patients with WAVE3-positive expression often had poorer tumor differentiation and distant metastasis.

## MATERIALS AND METHODS

### Animal

Athymic nude mice (BALB/c-nu, female), 4 weeks old and weighing 14-16 g, were purchased from Nanjing Model Animal Research Center (MARC). Animal certification label: SCXK2010-0001 (China). All animals were provided sterilized food and water. All experiments were carried out in accordance with the guidelines on animal care and experiments of laboratory animals (Center of Experimental Animals, Nanjing University of technology, China), which was approved by the ethics committee for animal experiments.

### Cell culture and stable transfection

Human ovarian cancer cell lines SW626, OVCAR-3 and SKOV3 were purchased from ATCC. The SW626 cell line was cultured in Leibowitz's L-15 Medium supplemented with 10% FBS. The SKOV3 cell line was cultured in McCoy's 5a Modified Medium supplemented with 10% FBS. The human ovarian cancer cell lines OVCAR-3, A2780 and A2780/Taxol (A2780/T), a Taxol-resistant cell line (Enjing Biotech) were cultured in RPMI 1640 medium supplemented with 10% FBS (GIBCO, California, USA) and 1% penicillin/streptomycin. To generate stable WAVE3 knockdown cells, low passage SKOV3 cells were seeded in 6-well culture plate one day before transfection, and then were stably transfected with human WAVE3 shRNA lentiviruses or non-targeting control lentiviruses (abm) using Lipofectamine 2000 (Invitrogen) according to the manufacturer's instruction. After 48 hours, cells were selected in RPMI 1640 medium containing 300 μg/mL G418. Stable WAVE3 high-expression cells were generated in similar way. Briefly, A2780 cells were transfected with human pcDNA3.1-WAVE3 plasmid or non-targeting control plasmid using EnoGeneFec 2000 transfection reagent (EnoGene) according to the manufacturer's instruction, and selected in RPMI 1640 medium containing 300 μg/mL G418. Stably expressed strains were acquired by expanded culture and further confirmed by quantitative real-time PCR and western blotting for WAVE3 expression.

### Cell growth curve

1 × 10^5^ cells suspended in medium with 10% FBS were added to 96-well plates, every cell line has five duplicate well. In next 5 days, cells in each well were counted under light microscope. On day 5, each well was added 5 μL CCK8 solution (EnoGene). After incubation in 37°C for 2 h, the absorbance was recorded at 450 nm with a 96-well plate reader (Thermo Fisher).

### Cell migration assay

Cell migration was measured according to the ability of the cells to migrate across a transwell filter (8 μm pores, Costar, Cambridge, MA, USA). 1 × 10^5^ cells suspended in serum-free medium were added to the upper chamber and medium containing 10 % FBS was added to the lower chamber. After incubation in 37°C for 24 h, the non-migrated cells were scraped off the filter using a cotton swab. The cells that migrated to the lower side from the upper chamber were fixed with 4 % paraformaldehyde and stained with hematoxylin. The cells per microscopic field were taken pictures (20×) and counted in 8 randomly chosen fields.

### Cell cycle analysis

Cells (5×10^5^) were harvested and washed twice with PBS. Cell samples were resuspended and fixed with 1 mL cold 70% ethanol at 4°C overnight. After washed and resuspended in 1 mL PBS for 15 min at room temperature, the fixed cells were incubated with 100 μL RNase A at 37˚C for 30 min followed by staining with 400 μL propidium iodide (PI) solution for 30 min in the dark. Cell cycle of samples was analyzed on FACSCalibur (BD Biosciences). The data were analyzed using FlowJo software.

### Cell apoptosis analysis

Annexin V/PI double staining and flow cytometry were used to analyze cell apoptosis. According to the instruction of cell apoptosis analysis kit (EnoGene), cells (5×10^5^) were harvested after trypsinized and centrifuged at 1500 rpm for 5 min. Cell samples were resuspened by 500 μL binding buffer and incubated with 5 μL AnnexinV-EGFP and 5 μL of PI in darkness for 15 min. 10000 cells per sample were collected and analyzed on FACSCalibur (BD Biosciences). The data were analyzed using FlowJo software.

### Quantitative real-time PCR

Total RNA was isolated from cells with TRIzol Reagent (Life Technologies, USA) according to the manufacturer's protocol, which was further reverse-transcribed to cDNA with a RevertAid First Strand cDNA Synthesis Kit (Thermo Scientific). The resulting cDNA was amplified with the QuantiFast SYBR Green PCR Kit. The GAPDH gene was used as the housekeeping control against which to normalize the expression of the target genes. The assays were performed in the iCycler iQ™ Single Color Real-Time PCR Detection System (Bio-Rad Laboratories, Hercules, CA, USA). Fold induction of gene expression was calculated using the ΔΔCT method as described early [50]. The primers were synthesized by Invitrogen and the primer sequences were: GAPDH, 5’- CCTCTGACTTCAACAGCGACAC-3’ and 5’- CTGTTGCTGTAGCCAAATTCGT -3’, WAVE3, 5’- CACCAATCAGTGATGCTCGAAG -3’ and 5’- AGTCGGACCAGTCGTTCTCG -3’.

### Western blotting

After rinsed twice with PBS buffer, cell extracts were collected in RIPA lysis buffer supplemented with 10 mM PMSF and phosphatase inhibitor on ice for 30 min. Protein concentrations were determined using the BCA protein assay kit (EnoGene). 50 μg of total protein were separated by 8-10 % SDS–PAGE gels and electro-transferred onto polyvinylidene difluoride membranes which were blocked with 5% skim milk (w/v) at room temperature for 60 minutes. The membranes were then incubated with primary antibodies recognizing MMP2, MMP9, NF-κB, COX2, VEGF (EnoGene), P38, p-P38, GAPDH (Cell Signaling Technology, Inc. Beverly, Mass, USA) and HRP-secondary antibody. The immunoreactive bands were developed using an ECL detection system. GAPDH was used as the loading control. For quantification, the grey density of target bands was analyzed by Image J software (National Institutes of Health, Bethesda, Maryland, U.S.) and normalized to the grey density of GAPDH.

### Immunohistochemistry (IHC) with human ovarian tissue

Tissue samples of ovarian were obtained from Department of Gynecological Oncology Surgery, Jiangsu Cancer Hospital & Institute, Nanjing Medical University. The presence of samples was verified by hematoxylin-eosin-stained section. The specimens were then reviewed, classified, and graded by two independent pathologists. All human ovarian tissues were taken from 60 adults, 20 of them were patients with ovarian cancer, 20 with advanced ovarian cancer and the remaining 20 were normal ovarian tissue from endometrial carcinoma after surgery. The study was conducted in accordance with the declaration of Helsinki and was approved by the ethics committee at the Jiangsu Cancer Hospital. The tissues were fixed for 24 h in 10% neutral buffered formalin, dehydrated them for 24 h in 70% ethanol and embedded in paraffin. For immunohistochemical analysis, we prepared 4-mm sections from paraffin-embedded tissue and investigated their reactivity to the WAVE3 antibody (EnoGene). We detected a specific signal using the appropriate DAB kit (Beyotime).

### Animal xenograft analysis

Four to five-weeks old BALB/c athymic nude mice were used for xenograft tumor studies. SKOV3/shRNA-WAVE3 stable cells, A2780/ pcDNA 3.1-WAVE3 stable cells and corresponding control cells were harvested in the exponential growth phase and washed twice with PBS, and resuspended in PBS/matrigel (1:1) at the dose of 5 × 10^6^ per 200 μL. 200 μL of cells were injected subcutaneously into the right armpit of nude mice. After tumor inoculation, tumor growth speed and tumor size were examined for 3 weeks, and tumors on the 21th day were removed and weighted. The growth of primary tumors was monitored once in two days by measuring tumor diameters with calipers and calculating tumor volume using the formula: volume=width^2^×length/2.

### Statistical analysis

The data were expressed as mean ± standard deviation (SD). The significance of the results obtained from the control and ovarian cancer groups was performed by Student's unpaired *t*-test and one way analysis of variance (ANOVA), further graphed with GraphPad Prism 5 (GraphPad Software, San Diego, CA, USA). Statistical significance was set as two-tailed P value less than 0.05. Analyses were performed with SPSS 17.0 software (Chicago, IL, United States).

## Abbreviations

CCK: Cell Counting Kit
COX: cyclooxygenase
DAB: diaminobenzidine
ECM: extracellular matrix
FBS: Fetal Bovine Serum
G148: Geneticin148
GAPDH: glyceraldehyde-phosphate dehydrogenase
IHC: Immunohistochemistry
MAPK: Mitogen-activated protein kinase
MARC: Model Animal Research Center
MMPs: Matrix metalloproteinases
NCI: National Cancer Institute’s
PBS: phosphate buffer saline
PI: propidium iodide
RPMI: Roswell Park Memorial Institute
siRNA: Small interfering RNA
TNM: Tumor Node Metastasis
VEGF: vascular endothelial growth factor
WAVE: Wiskott-Aldrich syndrome verprolin-homologous
WASP: Wiskott-Aldrich syndrome family proteins

## References

[1] Tingulstad S, Skjeldestad FE, Halvorsen TB, Hagen B (2003). Survival and prognostic factors in patients with ovarian cancer. Obstet Gynecol.

[2] Ozols RF (2005). Treatment goals in ovarian cancer. Int J Gynecol Cancer.

[3] Hanahan D, Weinberg RA (2000). The hallmarks of cancer. Cell.

[4] Kurisu S, Takenawa T (2009). The WASP and WAVE family proteins. Genome Biol.

[5] Caldieri G, Ayala I, Attanasio F, Buccione R (2009). Cell and molecular biology of invadopodia. Int Rev Cell Mol Biol.

[6] Kurisu S, Takenawa T (2010). WASP and WAVE family proteins: friends or foes in cancer invasion?. Cancer Sci.

[7] Sossey-Alaoui K, Li X, Ranalli TA, Cowell JK (2005). WAVE3-mediated cell migration and lamellipodia formation are regulated downstream of phosphatidylinositol 3-kinase. J Biol Chem.

[8] Sossey-Alaoui K, Ranalli TA, Li X, Bakin AV, Cowell JK (2005). WAVE3 promotes cell motility and invasion through the regulation of MMP-1, MMP-3, and MMP-9 expression. Exp Cell Res.

[9] Sossey-Alaoui K, Safina A, Li X, Vaughan MM, Hicks DG, Bakin AV, Cowell JK (2007). Down-regulation of WAVE3, a metastasis promoter gene, inhibits invasion and metastasis of breast cancer cells. Am J Pathol.

[10] Moazzam M, Ye L, Sun PH, Kynaston H, Jiang WG (2015). Knockdown of WAVE3 impairs HGF induced migration and invasion of prostate cancer cells. Cancer Cell Int.

[11] Ji Y, Li B, Zhu Z, Guo X, He W, Fan Z, Zhang W (2015). Overexpression of WAVE3 promotes tumor invasiveness and confers an unfavorable prognosis in human hepatocellular carcinoma. Biomed Pharmacother.

[12] Zhang Y, Guan XY, Dong B, Zhao M, Wu JH, Tian XY, Hao CY (2012). Expression of MMP-9 and WAVE3 in colorectal cancer and its relationship to clinicopathological features. J Cancer Res Clin Oncol.

[13] Sossey-Alaoui K (2013). Surfing the big WAVE: Insights into the role of WAVE3 as a driving force in cancer progression and metastasis. Semin Cell Dev Biol.

[14] Kulkarni S, Augoff K, Rivera L, McCue B, Khoury T, Groman A, Zhang L, Tian L, Sossey-Alaoui K (2012). Increased expression levels of WAVE3 are associated with the progression and metastasis of triple negative breast cancer. PLoS One.

[15] Taylor MA, Davuluri G, Parvani JG, Schiemann BJ, Wendt MK, Plow EF, Schiemann WP, Sossey-Alaoui K (2013). Upregulated WAVE3 expression is essential for TGF-beta-mediated EMT and metastasis of triple-negative breast cancer cells. Breast Cancer Res Treat.

[16] Sossey-Alaoui K, Downs-Kelly E, Das M, Izem L, Tubbs R, Plow EF (2011). WAVE3, an actin remodeling protein, is regulated by the metastasis suppressor microRNA, miR-31, during the invasion-metastasis cascade. Int J Cancer.

[17] Fernando HS, Sanders AJ, Kynaston HG, Jiang WG (2010). WAVE3 is associated with invasiveness in prostate cancer cells. Urol Oncol.

[18] Kessenbrock K, Plaks V, Werb Z (2010). Matrix metalloproteinases: regulators of the tumor microenvironment. Cell.

[19] Roy R, Yang J, Moses MA (2009). Matrix metalloproteinases as novel biomarkers and potential therapeutic targets in human cancer. J Clin Oncol.

[20] Chakraborti S, Mandal M, Das S, Mandal A, Chakraborti T (2003). Regulation of matrix metalloproteinases: an overview. Mol Cell Biochem.

[21] Coussens LM, Werb Z (1996). Matrix metalloproteinases and the development of cancer. Chem Biol.

[22] Fernando HS, Sanders AJ, Kynaston HG, Jiang WG (2008). WAVE1 is associated with invasiveness and growth of prostate cancer cells. J Urol.

[23] Suetsugu S, Yamazaki D, Kurisu S, Takenawa T (2003). Differential roles of WAVE1 and WAVE2 in dorsal and peripheral ruffle formation for fibroblast cell migration. Dev Cell.

[24] Kurisu S, Suetsugu S, Yamazaki D, Yamaguchi H, Takenawa T (2005). Rac-WAVE2 signaling is involved in the invasive and metastatic phenotypes of murine melanoma cells. Oncogene.

[25] Martin TA, Pereira G, Watkins G, Mansel RE, Jiang WG (2008). N-WASP is a putative tumour suppressor in breast cancer cells, *in vitro* and *in vivo*, and is associated with clinical outcome in patients with breast cancer. Clin Exp Metastasis.

[26] Sossey-Alaoui K, Bialkowska K, Plow EF (2009). The miR200 family of microRNAs regulates WAVE3-dependent cancer cell invasion. J Biol Chem.

[27] Miki H, Fukuda M, Nishida E, Takenawa T (1999). Phosphorylation of WAVE downstream of mitogen-activated protein kinase signaling. J Biol Chem.

[28] Coso OA, Chiariello M, Yu JC, Teramoto H, Crespo P, Xu N, Miki T, Gutkind JS (1995). The small GTP-binding proteins Rac1 and Cdc42 regulate the activity of the JNK/SAPK signaling pathway. Cell.

[29] Minden A, Karin M (1997). Regulation and function of the JNK subgroup of MAP kinases. Biochim Biophys Acta.

[30] Ricote M, Garcia-Tunon I, Bethencourt F, Fraile B, Onsurbe P, Paniagua R, Royuela M (2006). The p38 transduction pathway in prostatic neoplasia. J Pathol.

[31] Recio JA, Merlino G (2002). Hepatocyte growth factor/scatter factor activates proliferation in melanoma cells through p38 MAPK, ATF-2 and cyclin D1. Oncogene.

[32] Bulavin DV, Furnace AJ (2004). p38 MAP kinase's emerging role as a tumor suppressor. Adv Cancer Res.

[33] Hui L, Bakiri L, Stepniak E, Wagner EF (2007). p38alpha: a suppressor of cell proliferation and tumorigenesis. Cell Cycle.

[34] Ambrosino C, Nebreda AR (2001). Cell cycle regulation by p38 MAP kinases. Biol Cell.

[35] Ou Y, Zheng X, Gao Y, Shu M, Leng T, Li Y, Yin W, Zhu W, Huang Y, Zhou Y, Tang J, Qiu P, Yan G (2014). Activation of cyclic AMP/PKA pathway inhibits bladder cancer cell invasion by targeting MAP4-dependent microtubule dynamics. Urol Oncol.

[36] Caino MC, Chae YC, Vaira V, Ferrero S, Nosotti M, Martin NM, Weeraratna A, O’Connell M, Jernigan D, Fatatis A, Languino LR, Bosari S, Altieri DC (2013). Metabolic stress regulates cytoskeletal dynamics and metastasis of cancer cells. J Clin Invest.

[37] Hanahan D, Weinberg RA (2011). Hallmarks of cancer: the next generation. Cell.

[38] Hahn WC, Stewart SA, Brooks MW, York SG, Eaton E, Kurachi A, Beijersbergen RL, Knoll JH, Meyerson M, Weinberg RA (1999). Inhibition of telomerase limits the growth of human cancer cells. Nat Med.

[39] Miki H, Suetsugu S, Takenawa T (1998). WAVE, a novel WASP-family protein involved in actin reorganization induced by Rac. EMBO J.

[40] Suetsugu S, Miki H, Takenawa T (1999). Identification of two human WAVE/SCAR homologues as general actin regulatory molecules which associate with the Arp2/3 complex. Biochem Biophys Res Commun.

[41] Zhang L, Shi J, Feng J, Klocker H, Lee C, Zhang J (2004). Type IV collagenase (matrix metalloproteinase-2 and -9) in prostate cancer. Prostate Cancer Prostatic Dis.

[42] Al-Alem L, Curry TE (2015). Ovarian cancer: involvement of the matrix metalloproteinases. Reproduction.

[43] Kunapuli P, Chitta KS, Cowell JK (2003). Suppression of the cell proliferation and invasion phenotypes in glioma cells by the LGI1 gene. Oncogene.

[44] Ahmad A, Biersack B, Li Y, Kong D, Bao B, Schobert R, Padhye SB, Sarkar FH (2013). Targeted regulation of PI3K/Akt/mTOR/NF-kappaB signaling by indole compounds and their derivatives: mechanistic details and biological implications for cancer therapy. Anticancer Agents Med Chem.

[45] Matsuo Y, Sawai H, Ochi N, Yasuda A, Sakamoto M, Takahashi H, Funahashi H, Takeyama H, Guha S (2010). Proteasome inhibitor MG132 inhibits angiogenesis in pancreatic cancer by blocking NF-kappaB activity. Dig Dis Sci.

[46] Wang YC, Wu YN, Wang SL, Lin QH, He MF, Liu QL, Wang JH (2016). Docosahexaenoic Acid Modulates Invasion and Metastasis of Human Ovarian Cancer via Multiple Molecular Pathways. Int J Gynecol Cancer.

[47] Masoumi Moghaddam S, Amini A, Morris DL, Pourgholami MH (2012). Significance of vascular endothelial growth factor in growth and peritoneal dissemination of ovarian cancer. Cancer Metastasis Rev.

[48] Issbrucker K, Marti HH, Hippenstiel S, Springmann G, Voswinckel R, Gaumann A, Breier G, Drexler HC, Suttorp N, Clauss M (2003). p38 MAP kinase--a molecular switch between VEGF-induced angiogenesis and vascular hyperpermeability. FASEB J.

[49] Belotti D, Calcagno C, Garofalo A, Caronia D, Riccardi E, Giavazzi R, Taraboletti G (2008). Vascular endothelial growth factor stimulates organ-specific host matrix metalloproteinase-9 expression and ovarian cancer invasion. Mol Cancer Res.

[50] Livak KJ, Schmittgen TD (2001). Analysis of relative gene expression data using real-time quantitative PCR and the 2(-Delta Delta C(T)) Method. Methods.

